# Redox regulation of hemodynamics response to diadenosine tetraphosphate an agonist of P2 receptors and renal function in diet‐induced hypercholesterolemic rats

**DOI:** 10.14814/phy2.14888

**Published:** 2021-06-10

**Authors:** Kamil Dąbkowski, Ewelina Kreft, Kornelia Sałaga‐Zaleska, Gabriela Chyła, Agnieszka Kuchta, Maciej Jankowski

**Affiliations:** ^1^ Department of Clinical Chemistry Medical University of Gdańsk Gdańsk Poland

**Keywords:** cortical and medullary renal blood flow, diadenosine tetraphosphate, hypercholesterolemia, kidney, purinergic receptors

## Abstract

Hypercholesterolemia and oxidative stress may lead to disturbances in the renal microvasculature in response to vasoactive agents, including P2 receptors (P2R) agonists. We investigated the renal microvascular response to diadenosine tetraphosphate (Ap_4_A), an agonist of P2R, in diet‐induced hypercholesteremic rats over 28 days, supplemented in the last 10 days with tempol (2 mM) or DL‐buthionine‐(S,R)‐sulfoximine (BSO, 20 mM) in the drinking water. Using laser Doppler flowmetry, renal blood perfusion in the cortex and medulla (CBP, MBP) was measured during the infusion of Ap_4_A. This induced a biphasic response in the CBP: a phase of rapid decrease was followed by one of rapid increase extended for 30 min in both the normocholesterolemic and hypercholesterolemic rats. The phase of decreased CBP was not affected by tempol or BSO in either group. Early and extended increases in CBP were prevented by tempol in the hypercholesterolemia rats, while, in the normocholesterolemic rats, only the extended increase in CBP was affected by tempol; BSO prevented extended increase in CBP in normocholesterolemic rats. MBP response is not affected by hypercholesterolemia. The hypercholesterolemic rats were characterized by increased urinary albumin and 8‐isoPGF_2α_ excretion. Moreover, BSO increased the urinary excretion of nephrin in the hypercholesterolemic rats but, similar to tempol, did not affect the excretion of albumin in their urine. The results suggest the important role of redox balance in the extracellular nucleotide regulation of the renal vasculature and glomerular injury in hypercholesterolemia.

## Graphical abstract

Tempol abolishes the late phase of the diadenosine tetraphosphate (Ap_4_A)‐induced increase in cortical blood perfusion (CBP) in normocholesterolemic rats and the early and late phases of the Ap_4_A‐induced increase in CBP in hypercholesterolemic rats. Tempol does not modify the Ap_4_A‐induced reduction of CBP in normocholesterolemic and hypercholesterolemic rats.

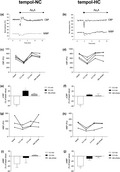

## 1 INTRODUCTION

Reactive oxide species (ROS) (e.g., superoxide anion, hydrogen peroxide) and reactive nitrogen species (RNS) (e.g., nitric oxide, peroxynitrite) affect renal vascular and tubular function under physiological conditions (Nistala et al., 2008). ROS/RNS are formed by enzymes, mainly NADPH oxidase, xanthine oxidase, cyclooxygenase, and nitric oxide synthase (Tejero et al., 2019), and are neutralized by an antioxidant system consisting of enzymes (e.g., superoxide dismutase, catalase, and glutathione [GSH] peroxidase), hydrophilic antioxidants (e.g., GSH), and lipophilic antioxidants (e.g., tocopherol) (He et al., 2017). Imbalance between the generation and utilization of ROS/RNS leads to oxidative stress that impairs the renal function and participates in kidney injury (Nistala et al., 2008; Wilcox, 2003). The biological trigger of redox stress is the superoxide forming reactive intermediates, which modifies proteins, DNA bases, and lipids via nitration, rendering the biomolecules dysfunctional (Pacher et al., 2007).

A growing body of evidence suggests that numerous pathological conditions, including hypercholesterolemia, are associated with an increased vascular production of ROS/RNS (Napoli & Lerman, 2001). An enhanced ROS/RNS production has also been demonstrated in hypercholesterolemic animal models (Ohara et al., 1993; Onody et al., 2003); however, the dietary correction of hypercholesterolemia normalizes endothelial ROS production (Ohara et al., 1995).

Hypercholesterolemia occurred in early arthrosclerosis is characterized by oxidative stress, endothelial dysfunction, and inflammation, impairing the renal vascular function in vitro (Cachofeiro et al., 2008; Stulak et al., 2001). Diet‐induced hypercholesterolemia is associated with enhanced glomerular capillary pressure following an increased glomerular filtration rate preceding the development of chronic kidney disease (Kasiske et al., 1990). Hypercholesterolemia in rats aggravates the renal injury primarily via podocyte damage with the activation of focal adhesion kinase and cytoskeleton reorganization (Hu et al., 2017; Joles et al., 2000), decreases renal cortical nitric oxide synthesis, and induces podocyte stress (Attia et al., 2004). Podocytes are key cells for the maintenance of the permeability properties of the glomerular filtration barrier, and their defect can lead to an increased amount of proteins, including albumin, passing through the glomerular filter and then flowing into the urine and affecting the function of the tubular cells (Guo et al., 2012). It has been shown that albuminuria is associated with hypercholesterolemia (Shankar et al., 2004).

Extracellular nucleotides and dinucleotide polyphosphates have been shown to exert powerful physiological effects on renal hemodynamics and its function following their activation of P2 receptors (P2Rs) divided into ionotropic ligand‐gated P2X and G protein‐coupled receptors. The distribution‐ and expression‐specific P2R subtypes are ubiquitous throughout the body, with distributions varying regionally and overlapping across tissues, including the kidneys in both the cortical and medullary vascular and tubular compartments (Kadiiska et al., 2005). P2Rs contribute to a diverse range of (patho)physiological processes, including cortical and medullary renal blood flows, glomerular filtration and permeability to albumin, control of renin release, and the regulation of renal tubular transport. Extracellular ATP activates NADPH oxidase in podocytes, which leads to redox stress (Greka & Mundel, 2012). On the other hand, hypercholesterolemia affects the metabolism of extracellular nucleotides, because their hydrolysis is enhanced in the platelets of patients with hypercholesterolemia (Onody et al., 2003). Interestingly, genetic defects in the receptors for extracellular nucleotides are associated with atherosclerosis‐dependent myocardial infarction (Wang et al., 2009).

Significant amounts of dinucleoside polyphosphates, mainly diadenosine tetraphosphate (Ap_4_A), are stored in platelet‐dense granules and are released into the blood after platelet activation (Jankowski et al., 2009). The mean human plasma concentration of Ap_n_A (*n* = 3–6) is in the range of 10–40 nM, with the highest concentrations achieved by Ap_4_A (Schulz et al., 2014). In the extracellular space, diadenosine polyphosphates interact with P2Rs and are metabolized by enzymes possessing ecto‐Ap_n_A hydrolase activity (Drygalski & Ogilvie, 2000). Ap_4_A affects both renal microvasculature, including the glomerular capillary network (Szczepanska‐Konkel et al., 2005) and renal tubular function (Stiepanow‐Trzeciak et al., 2007). The renal hemodynamic response to Ap_4_A is modified under hyperglycemic conditions (Kreft et al., 2020) accompanied by redox stress (Jha et al., 2016).

Despite a large body of evidence demonstrating the injurious effect of hypercholesterolemia on the systemic and coronary vasculatures, data pertaining to its effects on the renal vasculature and the modification of the vascular response to agonists of P2R is rather limited. Hence, we have measured in vivo changes in cortical and medullary blood perfusion (MBP) due to Ap_4_A action in rats fed a hypercholesterolemic diet (HC) wherein further modified redox state by administering tempol, a scavenger of the superoxide anion, and DL‐buthionine‐(S,R)‐sulfoximine (BSO), an inductor of systemic oxidative stress. Additionally, the urinary excretion of oxidative stress markers and specific proteins were measured.

## 2 MATERIALS AND METHODS

### 2.1 Ethical approval

The experiments were conducted in accordance with the European Convention for the Protection of Vertebrate Animals used for Experimental and other Scientific Purposes and approved by the local Bioethics Commission in Bydgoszcz, Poland (8/2017, 23/2019).

### 2.2 Animals

The experiments were performed on male Wistar rats (200–250 g) purchased from Tri‐City Academic Laboratory Animal Centre—Research and Services Centre (Medical University of Gdansk) and housed under a 12‐h light/dark cycle at an ambient temperature of 22°C with free access to food and tap water.

### 2.3 Experimental protocol

The rats were treated for 4 weeks. Six groups of rats were studied, each group consisting of 9–10 animals. The first three groups (I–III) received a normocholesterolemic diet (NC) (Labofeed B, Zofia Połczyńska Wytwórnia Pasz "Morawski") containing 20% digestible protein, and the second three groups (IV–VI) were fed a standard diet to which 2% cholesterol and 0.5% sodium cholate had been added, HC (Zofia Połczyńska Wytwórnia Pasz "Morawski"). The different groups received the following in their drinking water in the last 10 days:

1. Groups I (NC) and IV (HC): water.
2. Groups II (NC) and V (HC): 2 mM tempol.
3. Groups III (NC) and VI (HC): 20 mM BSO.

The renal blood perfusion measurements were performed on three to four anesthetized rats from each group.

### 2.4 Renal blood perfusion measurement

The rats were anesthetized with an i.p. injection of Inactin^™^ (i.p., 100 mg/kg), and a polyethylene tube was placed in the trachea to ensure a free airway. The animals were kept on a thermostatically controlled heated table at 37°C. Polyethylene catheters (PE‐50) were inserted into the femoral vein for saline infusion (World Precision Instruments) and into the femoral artery to permit periodic blood sampling and the continuous monitoring of the mean arterial blood pressure, which was recorded using a catheter filled with heparinized (30 IU/ml) isotonic saline solution and connected to a pressure transducer (MP 150; Biopac Systems Inc.). The rats received intravenous 0.9% NaCl (an injection of 200 µl and then an infusion at a rate of 45 µl/min) containing 1 IU/ml heparin. The urinary bladder was exposed through an abdominal incision and catheterized. The left kidney was exposed by a subcostal flank incision and placed in a Lucite^™^ holder. Renal blood perfusion was measured via laser Doppler flowmetry, LDF (Stern et al., 1979) based on the Doppler frequency shift that occurs when monochromatic laser light is scattered by a moving object. To measure the renal cortical blood perfusion (CBP), a PF 407 probe (Perimed AB) was placed on the kidney surface, and, to measure the MBP, a PF 402 probe (Perimed AB) was inserted into the kidney at a depth of 2–3 mm. The LDF signal sampled at the 32 Hz frequency was recorded continuously by an interfaced computer equipped with Perisoft dedicated software (Perimed). The LDF values were expressed in arbitrary perfusion units (PU). The animals were allowed to recover, as defined by stable hemodynamics and urinary flow rate, after the surgical preparation. After the recovery period, the experimental infusion of saline for 30 min was initiated. Next, each rat received Ap_4_A (an injection of 2 µmol/kg and then an infusion of 20 nmol/kg/min) for the next 30 min. The renal blood perfusion values derived from the first three 10‐min collection periods during the saline infusion were used as basal values. The average values of the blood perfusion recorded within the first and second 2‐min periods of Ap_4_A infusion were taken as the early phase 0–2 min and 2–4 min values, respectively. The blood perfusion values derived from the last 10‐min period of the 30‐min Ap_4_A infusion were taken as the late phase values. Upon completion of the experiment, all animals were sacrificed by means of maximum volume blood sampling via cardiopunction, and the position of the PF 402 probe in the renal medulla was checked.

### 2.5 Metabolic characterization

Total cholesterol (Pointe Scientific), triacylglycerols (Wiener Lab), and phospholipids (Wako Diagnostics) were measured via standard enzymatic colorimetric tests on a Multiskan^™^ GO Microplate Spectrophotometer (Thermo Fisher Scientific). High density lipoproteins (HDLs) were isolated via the precipitation of apolipoprotein B (apoB)‐containing lipoproteins with a heparin 5000/Mn^2+^ reagent, and HDL cholesterol (HDL‐C) was determined enzymatically (Warnick & Albers, 1978). Non‐HDL cholesterol (nonHDL‐C) was calculated according to the equation: nonHDL‐C = TC−HDL‐C. Thiobarbituric acid‐reactive substances (TBARS) and total GSH were measured using spectrophotometric methods (Rahman et al., 2006; Yokode et al., 1988). Enzymatic methods utilizing creatiniase (Pointe Scientific) have been used to measure creatinine concentration in the serum and urine (Moss et al., 1975). The glomerular filtration rate was calculated based on the clearance of creatinine. The urine total protein concentrations were measured using the modified Lowry method. Enzyme‐linked immunosorbent assay kits were used to measured rat oxLDL (Biorbyt Ltd, orb411300), 8‐OHdG (Cayman Chemical, kit 589320–96), rat nephrin (Wuhan EIAab Science Co., Ltd, E0937r), rat albumin (AssayPro, ERA3201‐1), and 8‐isoPGF_2α_ (Wuhan Fine Biotech Co., Ltd, ER1580). Urine metabolites and protein excretion were measured in samples obtained by housing the rats individually for 24 h in metabolic cages. Urine volume was determined gravimetrically.

### 2.6 Statistical analysis

The statistical analyses were performed using Sigma Plot 14.0 software. The Shapiro–Wilk test was used to test the determined normality of the distribution of the variables. Continuous variables with a normal distribution were reported as mean ± standard error for parametric data distribution or as median (IQR, 25th–75th percentile) for non‐parametric data distribution. Statistical significance was measured with a one‐way ANOVA and Holm‐Sidak post‐hoc test, a Welch ANOVA and Games‐Howell post‐hoc or a Kruskal Wallis test and Dunn post hoc test. *p* values below 0.05 were considered to be statistically significant.

### 2.7 Materials

Ap_4_A, Inactin^®^ hydrate, tempol were purchased from Merck KGaA. BSO was obtained from Acros Organics, and heparin from Polfa S.A. All other agents were purchased from Avantor Performance Material Poland S.A.

## 3 RESULTS

Table 1 summarizes the growth parameters (initial and final body weights, food intake) and water balance parameters (water intake, diuresis, and creatinine clearance) of the Wistar rats after 28 days of feeding on NC or HC diets. There were no significant differences in terms of body weight gain or the other parameters between the experimental groups. Tempol (2 mM), a superoxide dismutase mimetic, and BSO (20 mM), an irreversible inhibitor of gamma‐glutamylcysteine synthetase, were administrated to the drinking water to modify the level of oxidative stress (Ford et al., 2006; Welch et al., 2005). Tempol did not affect the growth parameters or water balance parameters in the NC and HC groups, but diuresis was about 30% lower in the NC group treated with BSO.

**TABLE 1:** Summary of 24‐h urine collection (metabolic cages) in rats fed NC or HC over 28 days without (water) and with the addition of tempol (2 mM) or BSO (20 mM) in the drinking water within the last 10 days of the experiment

	Body weight (g)		Food intake (g/24 h)		Water intake (ml/24 h)		Diuresis (ml/24 h)		Creatinine clearance (ml/min/100 g b.w.)
	Day 1	Day 29	Day 1	Day 29	Day 1	Day 29	Day 1	Day 29	Day 29
NC									
Water (*N* = 9)	279 ± 4	358 ± 9**	20.8 ± 0.2	22.4 ± 0.9	25 ± 1	27 ± 1	9 ± 1	12 ± 1	0.68 ± 0.05
Tempol (*N* = 9)	277 ± 5	364 ± 8**	20.8 ± 0.5	21.7 ± 0.9	26 ± 1	27 ± 2	9 ± 1	11 ± 1	0.62 ± 0.03
BSO (*N* = 9)	271 ± 5	340 ± 4**	22.3 ± 0.7	20.7 ± 0.8	28 ± 3	24 ± 2	9 ± 1	8 ± 1*	0.67 ± 0.04
HC									
Water (*N* = 10)	253 ± 7*	335 ± 11**	21.6 ± 0.5	22.6 ± 1.2	22 ± 2	26 ± 2	9 ± 1	9 ± 1	0.74 ± 0.02
Tempol (*N* = 10)	263 ± 5	341 ± 7**	22.2 ± 1.0	20.3 ± 0.8	23 ± 1	24 ± 1	10 ± 1	10 ± 1	0.68 ± 0.04
BSO (*N* = 10)	271 ± 2	341 ± 5**	22.4 ± 1.5	21.2 ± 0.8	23 ± 1	29 ± 1	9 ± 2	9 ± 1	0.65 ± 0.03

Results are expressed as mean ± SE. Statistical significance were measured using Welch ANOVA with a post‐hoc Games‐Howell test.

Abbreviations: BSO, DL‐buthionine‐(S,R)‐sulfoximine; HC, hypercholesterolemic diet; *N*, number of rats; NC, normocholesterolemic diet.

* *p* < 0.05 versus water NC.

** *p* < 0.0001 versus appropriate parameter on day 1.

Table 2 presents the lipid parameters in the serum of rats fed the NC or HC diets and the effects of tempol or BSO on these parameters. Feeding the rats with the HC diet led to increased concentrations of total cholesterol (three‐fold) and non‐HDL cholesterol (eight‐fold) as well as a 40% decrease in the HDL concentration. Additionally, the cholesterol treatment did not affect the concentrations of the serum triglycerides, phospholipids, or oxidized low density lipoproteins (oxLDLs). Furthermore, the modulators of oxidative stress did not affect the lipid parameter levels in either rat diet groups.

**TABLE 2:** Serum lipid profile in rats fed NC or HC over 28 days without (water) and with the addition of tempol (2 mM) or BSO (20 mM) in the drinking water within the last 10 days of the experiment

	Total cholesterol (mg/dl)	HDL cholesterol (mg/dl)	Non‐HDL cholesterol (mg/dl)	Triglycerides (mg/dl)	Phospholipids (mg/dl)	oxLDL (ng/ml)
NC						
Water (*N* = 9)	62 ± 4	42 ± 3	20 ± 3	110 ± 21	122 ± 10	21.85 ± 1.56
Tempol (*N* = 9)	60 ± 3	39 ± 2	21 ± 2	131 ± 19	129 ± 6	21.01 ± 1.13
BSO (*N* = 9)	64 ± 3	35 ± 4	28 ± 4	88 ± 12	115 ± 6	20.65 ± 1.33
HC						
Water (*N* = 10)	191 ± 19*	25 ± 2	166 ± 20*	94 ± 21	140 ± 6	20.27 ± 1.64
Tempol (*N* = 10)	174 ± 15*	25 ± 3	149 ± 14*	101 ± 17	134 ± 8	18.04 ± 0.85
BSO (*N* = 10)	200 ± 16*	25 ± 3	176 ± 17*	92 ± 18	133 ± 7	18.49 ± 1.39

Results are expressed as mean ± SE. Statistical significance were measured using one way ANOVA with post‐hoc Tukey method.

Abbreviations: BSO, DL‐buthionine‐(S,R)‐sulfoximine; HC, hypercholesterolemic diet; NC, normocholesterolemic diet.

* *p* < 0.001 in comparison to analogue NC.

The renal vascular responses, measured as the changes in CBP and MBP in response to the intravenous infusion of Ap_4_A, an agonist of P2R, were measured in rats fed the NC or HC diets either with the addition of tempol (tempol‐NC or tempol‐HC) or BSO (BSO‐NC or BSO‐HC) in the drinking water or without (water‐NC or water‐HC). CBP and MBP changes in the response to Ap_4_A were variable over the experimental period. For the purposes of the present study, two renal perfusion response phases were distinguished: an early phase of up to 4 min consisting of two subphases, each lasting up to 2 min after the start of Ap_4_A infusion, and a late phase, including the last 10 min of the 30‐min period from the start of Ap_4_A infusion.

**FIGURE 1:**
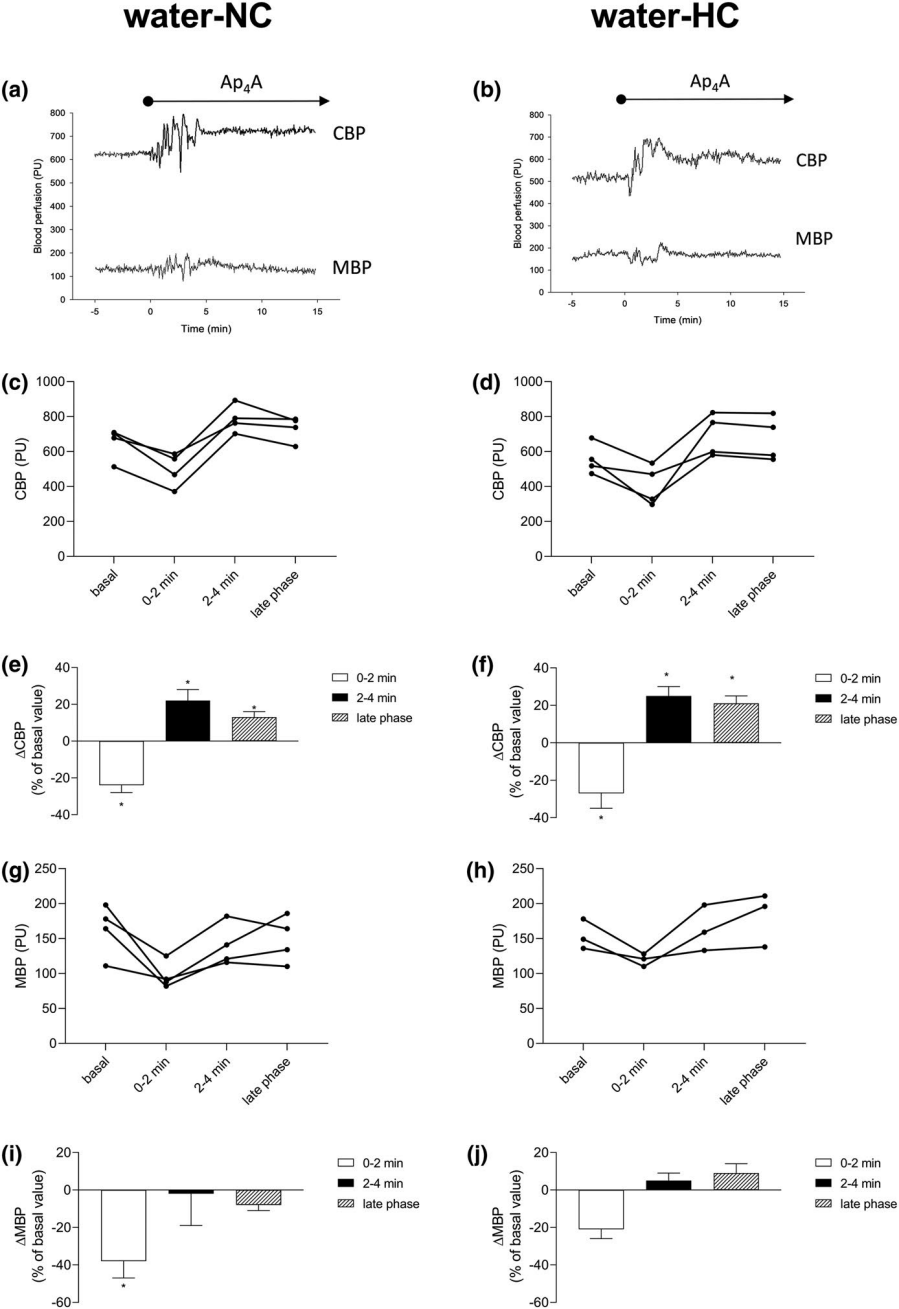
Ap_4_A affects renal blood perfusion in rats fed normocholesterolemic or hypercholesterolemic diets. The experiments were performed administering normocholesterolemic (water‐NC: a, c, e, g, i) and hypercholesterolemic (water‐HC: b, d, f, h, j) diets for 28 days. Original recordings of cortical (CBP) and medullary (MBP) blood perfusions during intravenous infusion of Ap_4_A at dose of 2 µmol/kg +20 nmol/kg/min (a, b). Individual results of CBP (c, d) and MBP (g, h) in rats fed normocholesterolemic (*n* = 4) and hypercholesterolemic (*n* = 3–4) diets; basal values are derived from three 10‐min periods of 0.9% NaCl infusion, average values recorded within first 2 min of Ap_4_A infusion (0–2 min) and within second 2 min of Ap_4_A infusion (2–4 min), values derived from last 10‐min period of 30‐min Ap_4_A infusion (late phase). The percentage of CBP (e, f) and MBP (i, j) changes during Ap_4_A infusion. The results are presented as mean ± SE. Statistical significance was measured using one‐way repeated measures ANOVA with a Holm‐Sidak post‐hoc test, *p < 0.05 versus basal value

The effects of Ap_4_A on the renal perfusion in the water‐NC or water‐HC groups are shown in Figure 1. The original recording of the CBP and MBP changes evoked by the Ap_4_A infusion in the individual rats from the water‐NC and water‐HC groups is presented in Figure 1a,b, respectively. Also, the individual CBP responses in the water‐NC or water‐HC groups are presented in Figure 1c,d, respectively. The early cortical perfusion responses in the water‐NC or water‐HC groups were dynamic and were characterized by a rapid decrease (0–2 min) followed by an increase (2–4 min). The average changes in CBP are presented in Figure 1e (water‐NC) and Figure 1f (water‐HC). The average value of basal CBP was 651 ± 46 PU in the water‐NC group. Ap_4_A induced a 24% decrease in CBP (496 ± 49 PU, *p* < 0.001) followed by a 22% increase (787 ± 40 PU, *p* = 0.001) in the water‐NC group (Figure 1e). Afterwards, the CBP values declined slightly; however, CBP in the late phase was maintained at a 13% higher level than the basal level (732 ± 36 PU, *p* = 0.022). In the water‐HC group, the average value of basal CBP was 556 ± 44 PU, and Ap_4_A induced a 27% decrease in CBP (407 ± 57 PU, *p* = 0.012) followed by a 25% increase (692 ± 57 PU, *p* = 0.017). In the late phase, CBP was maintained at a 21% higher level compare to the basal level (673 ± 63 PU, *p* = 0.034). The individual MBP responses in the water‐NC and water‐HC groups are presented in Figure 1g,h, respectively. The average values of basal MBP were 163 ± 17 PU (water‐NC) and 154 ± 12 PU (water‐HC). In the water‐NC Ap_4_A led to a decrease in MBP, with a subsequent return of MBP value to the basal level in the early phase. The average changes in MBP are presented in panel I (water‐NC) and panel J (water‐HC). In the water‐NC group (Figure 1i), Ap_4_A in the first 2 min induced a 38% decrease in MBP (97 ± 10 PU, *p* = 0.001), which was followed by a return to the basal value in the next 2 min (140 ± 15 PU, *p* = 0.143); MBP in the late phase was not significantly affected by Ap_4_A (148 ± 17 PU, *p* = 0.341). In the water‐HC group (Figure 1j), the reduction in MBP was about 21% within the first 2 min of Ap_4_A infusion (120 ± 5 PU) and fell short of statistical significance (*p* = 0.051). Afterwards, in next 2 min, the reduced MBP returned to the basal value (163 ± 19 PU, *p* = 0.103), and there was not a significant change in MBP in the later phase (182 ± 22 PU, *p* = 0.550).

**FIGURE 2:**
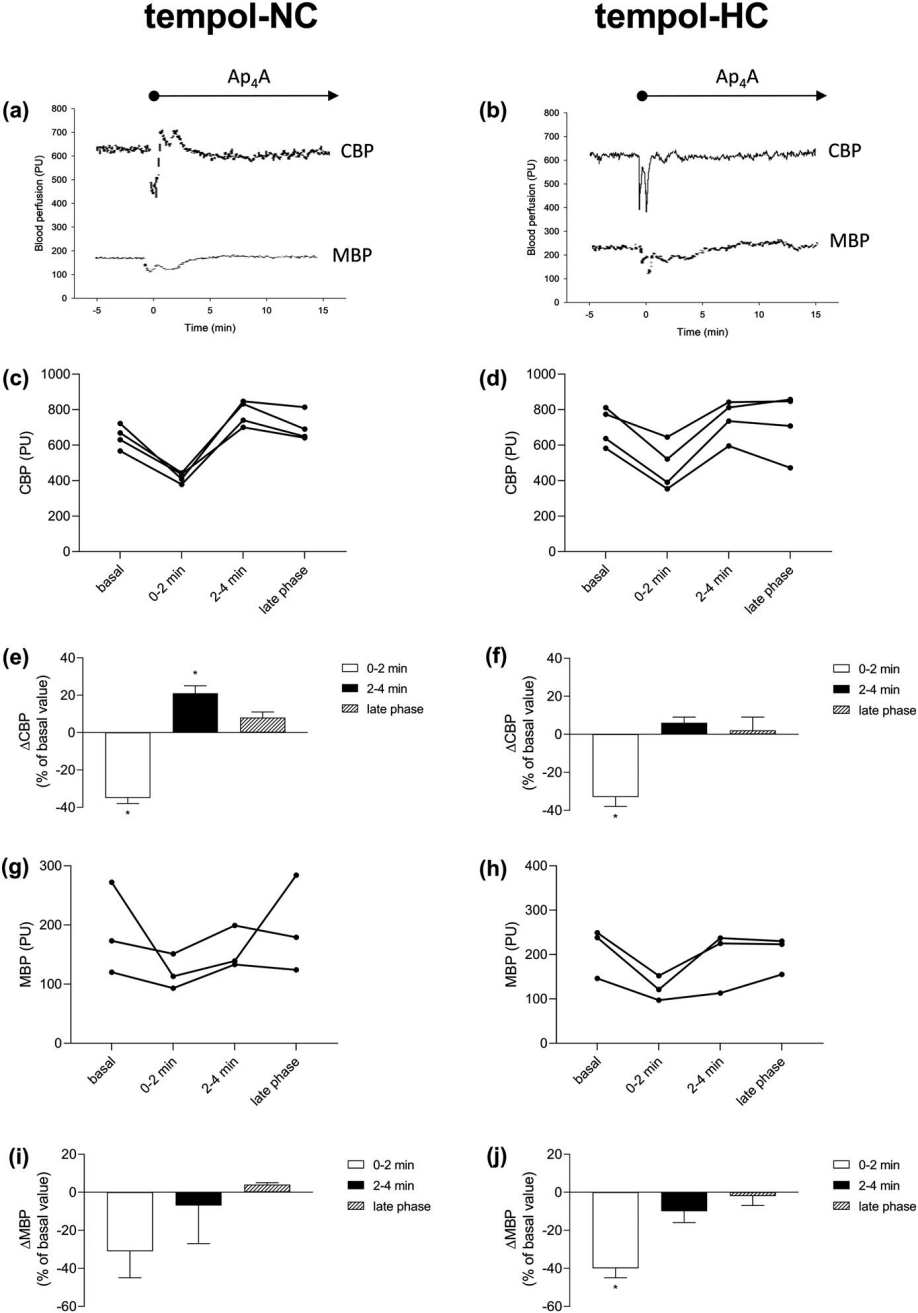
Ap_4_A affects renal blood perfusion in rats fed normocholesterolemic or hypercholesterolemic diets supplemented with tempol. The experiments were performed administering normocholesterolemic (tempol‐NC: a, c, e, g, i) or hypercholesterolemic (tempol‐HC: b, d, f, h, j) diets over 28 days supplemented with 2 mM tempol in the drinking water in the last 10 days. Original recordings of cortical (CBP) and medullary (MBP) blood perfusions during infusion of Ap_4_A at dose of 2 µmol/kg + 20 nmol/kg/min (a, b). Individual results of CBP (c, d) and MBP (g, h) in rats fed normocholesterolemic (*n* = 3–4) and hypercholesterolemic (*n* = 3–4) diets supplemented with tempol; basal values are derived from three 10‐min periods of 0.9% NaCl infusion, average values recorded within first 2 min of Ap_4_A infusion (0–2 min) and within second 2 min of Ap_4_A infusion (2–4 min), values derived from last 10‐min period of 30‐min Ap_4_A infusion (late phase). The percentage of CBP (e, f) and MBP (i, j) changes during Ap_4_A infusion. The results are presented as mean ± SE. Statistical significance was measured using one‐way repeated measures ANOVA with a Holm‐Sidak post‐hoc test, *p < 0.05 versus basal value

The effects of Ap_4_A on the cortical and MBPs in the rats fed the NC‐ and HC‐diets with the addition of tempol in their drinking water (2 mM, 10 days) are presented in Figure 2. The original recordings of the CBP and MBP responses to the Ap_4_A infusion in the individual rats from the tempol‐NC and tempol‐HC groups are presented in Figure 2a,b, respectively. Also, the individual responses of CBP in the tempol‐NC and tempol‐HC groups are presented in Figure 2c,d, respectively. The early cortical perfusion responses were dynamic with courses characterized by a rapid decrease (0–2 min) followed by an increase (2–4 min). The average changes in CBP are presented in Figure 2e (tempol‐NC) and Figure 2f (tempol‐HC).

The average value of basal CBP was 647 ± 33 PU in the tempol‐NC group; Ap_4_A induced a 35% decrease of CBP (417 ± 15 PU, *p* < 0.001) followed by a 21% increase (780 ± 36 PU, *p* = 0.002); afterwards, CBP declined to the basal value (688 ± 36 PU, *p* = 0.125). In the tempol‐HC group (Figure 2f), the basal CBP was 701 ± 54 PU, and Ap_4_A induced a 33% decrease in CBP (477 ± 66 PU, *p* < 0.001). In the next 2 min, CBP returned to the basal value (746 ± 55 PU, *p* = 0.262) and was maintained at the basal value up to the end of the experimental period (721 ± 90 PU, *p* = 0.606). The individual MBP responses in the tempol‐NC and tempol‐HC groups are presented in Figure 2g,h, respectively. The average values of basal MBP were 183 ± 44 PU (tempol‐NC) and 211 ± 33 PU (tempol‐HC). In the tempol‐NC group, Ap_4_A did not significantly affect MBP throughout the experimental period (*p* = 0.305); the average values of MBP for the experimental periods were: 123 ± 16 PU (first 2 min of Ap_4_A infusion), 191 ± 39 PU (second 2 min of Ap_4_A infusion), and 203 ± 24 PU (last 10 min of 30‐min period of Ap_4_A infusion). In the tempol‐HC group (Figure 2j), the reduction in MBP was about 40% within the first 2 min of Ap_4_A infusion (123 ± 16 PU, *p* = 0.003). Afterward, in the next 2 min, the reduced MBP returned to the basal value (192 ± 40 PU, *p* = 0.326), and there was not a significant change in the later phase (203 ± 24 PU, *p* = 0.666).

**FIGURE 3:**
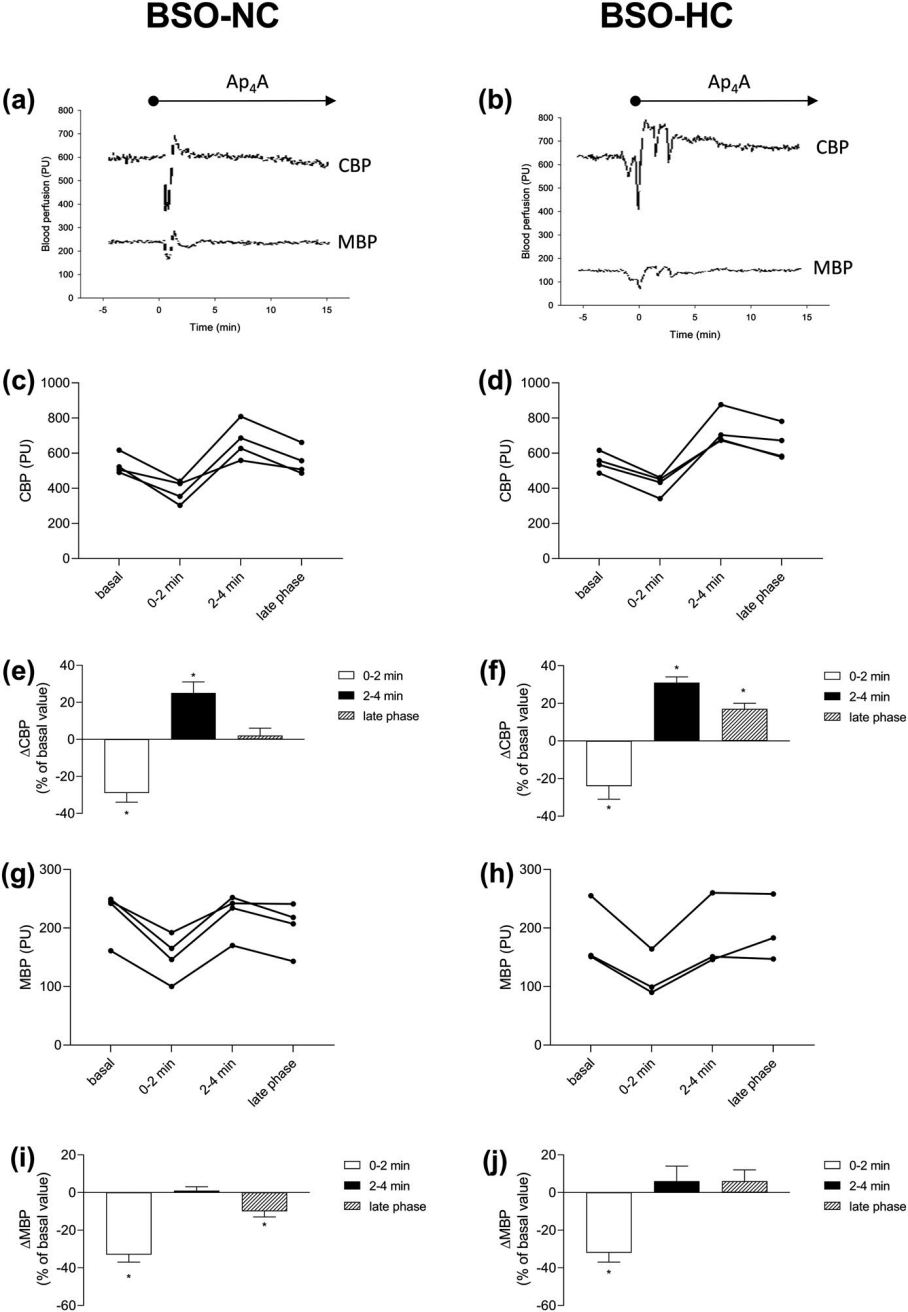
Ap_4_A affects renal blood perfusion in rats fed normocholesterolemic or hypercholesterolemic diets supplemented with BSO. The renal clearance experiments involved the administration of normocholesterolemic (BSO‐NC: a, c, e, g, i) or hypercholesterolemic (BSO‐HC: b, d, f, h, j) diets over 28 days supplemented with 20 mM BSO in (DL‐buthionine‐S,R‐sulfoximine) the drinking water in the last 10 days. Original recordings of cortical (CBP) and medullary (MBP) blood perfusions during infusion of Ap_4_A at dose of 2 µmol/kg + 20 nmol/kg/min (a, b). Individual results of CBP (c, d) and MBP (g, h) in rats fed normocholesterolemic (*n* = 4) and hypercholesterolemic (*n* = 3–4) diets supplemented with BSO; basal values are derived from three 10‐min periods of 0.9% NaCl infusion, average values recorded within first 2 min of Ap_4_A infusion (0–2 min) and within second 2 min of Ap_4_A infusion (2–4 min), values derived from last 10‐min period of 30‐min Ap_4_A infusion (late phase). The percentage of CBP (e, f) and MBP (i, j) changes during Ap_4_A infusion. The results are presented as mean ± SE. Statistical significance was measured using one‐way repeated measures ANOVA with a Holm‐Sidak post‐hoc test, *p < 0.05 versus basal value

The effects of Ap_4_A on cortical and MBP in the rats fed the NC‐ and HC‐diets with the addition of BSO in their drinking water (20 mM, 10 days) are presented in Figure 3. The original recordings of the CBP and MBP responses to the Ap4A infusion in the individual rats from the BSO‐NC and BSO‐HC groups are presented in Figure 3a,b, respectively. Also, the individual CBP responses in the rats from the BSO‐NC and BSO‐HC groups are presented in Figure 3c,d, respectively. The early cortical perfusion responses were dynamic, with courses characterized by a rapid decrease (0–2 min) followed by an increase (2–4 min) in values. The average changes in CBP are presented in Figure 3e (BSO‐NC) and Figure 3f (BSO‐HC). The average value of basal CBP was 534 ± 28 PU in the BSO‐NC group; Ap_4_A induced a 29% decrease (381 ± 32 PU, *p* = 0.002) followed by a 25% increase in CBP (670 ± 53 PU, *p* = 0.030). In the late phase, the CBP declined to the basal value (541 ± 29 PU, *p* = 0.831). The average value of basal CBP was 560 ± 39 PU in the BSO‐HC group; Ap_4_A induced a 24% decrease in basal CBP (422 ± 28 PU, *p* < 0.001), which was followed by a 31% increase (733 ± 48 PU, *p* = 0.004). Afterward, in the late phase, CBP values declined to reach a value 17% higher than the basal value (653 ± 48 PU, *p* = 0.029). The individual MBP responses in all rats in the BSO‐NC and BSO‐HC groups are presented in Figure 3g,h, respectively. The average values of basal MBP were 224 ± 21 PU (BSO‐NC) and 186 ± 34 PU (BSO‐HC). Ap_4_A led to a decrease in MBP with a subsequent return to the basal value in the early phase. The average changes in MBP are presented in Figure 3i (BSO‐NC) and Figure 3j (BSO‐HC). In the BSO‐NC group, Ap_4_A induced a 33% decrease in MBP (151 ± 19 PU, *p* < 0.001) in the first 2 min followed by a return to the basal value in the next 2 min (224 ± 18 PU, *p* = 0.964). The MBP value in the late phase was about 10% lower than basal value (201 ± 20 PU, *p* = 0.013). In the BSO‐HC group, the reduction in MBP was about 32% within the first 2 min of Ap_4_A infusion (117 ± 23 PU, *p* = 0.001). Afterwards, in next 2 min, the reduced MBP returned to the basal value (185 ± 33 PU, *p* = 0.419) and remained at a similar value in the later phase (196 ± 33 PU, *p* = 0.965).

**FIGURE 4:**
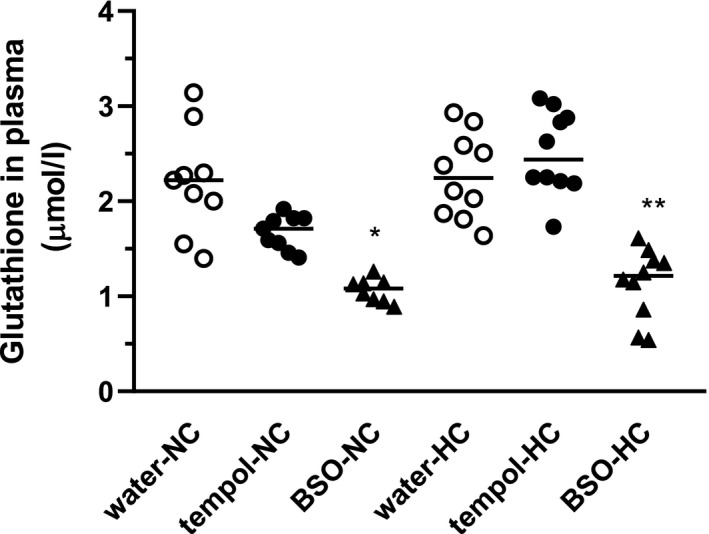
Effect of tempol and BSO on plasma total glutathione (GSH) concentration in rats fed normocholesterolemic or hypercholesterolemic diets. Rats were fed normocholesterolemic (NC) or hypercholesteremic (HC) diets over 28 days along with the oral supplementation (10 days) of water (○): 2 mM tempol (●) and 20 mM BSO (▲). The results are presented as individual data points with mean values. Statistical significance was measured using Welch ANOVA with a post‐hoc Games‐Howell post‐hoc test, *p = 0.0021 versus water‐NC, **p = 0.0001 versus water‐HC

For the determination of the effectiveness of BSO administration in inhibiting GSH synthesis, its concentrations in the plasma were measured (Figure 4). BSO treatment decreased GSH concentration by about 50% in the rats fed the NC‐diet (1.07 ± 0.04 nmol/L vs. 2.21 ± 0.19 nmol/L, *p* = 0.0021) and HC‐diet (1.14 ± 0.12 nmol/L vs 2.27 ± 0.14 nmol/L, *p* = 0.001).

**FIGURE 5:**
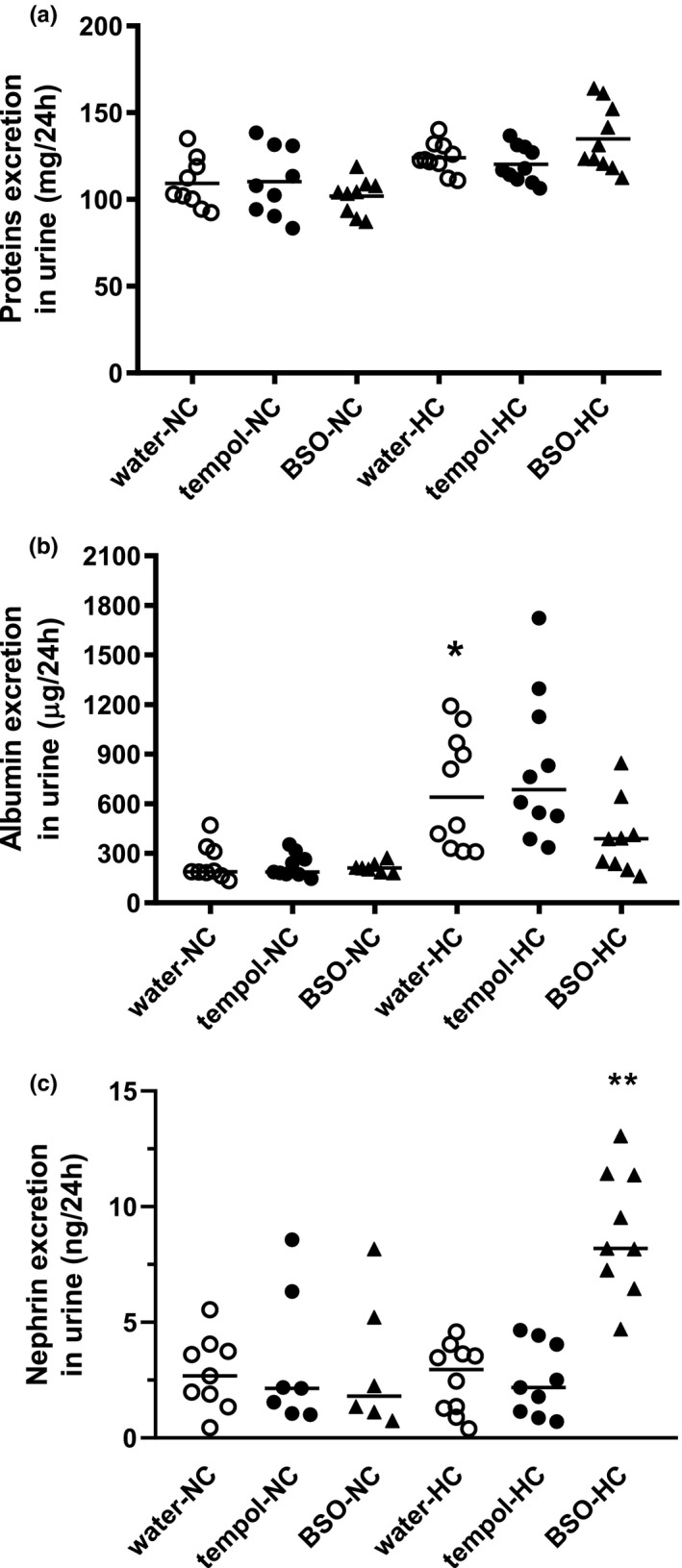
Excretion of urinary oxidative stress biomarkers is medicated by tempol and BSO in rats fed normocholesterolemic or hypercholesterolemic diets. Rats were fed normocholesterolemic (NC) or hypercholesteremic (HC) diets over 28 days along with the oral supplementation (10 days) of water (○): 2 mM tempol (●) and 20 mM BSO (▲). Urinary excretion of thiobarbituric acid reactive substances, TBARS (a), 8‐iso‐prostaglandin F_2α_, 8‐isoPGF_2α_ (b), 8‐hydroxy‐2'‐deoxyguanosine 8‐OHdG (c). The results are presented as individual data points with mean. Statistical significance was measured using Welch ANOVA with a post‐hoc Games‐Howell test, *p = 0.0196 versus water‐NC, **p = 0.031 versus water‐HC, ***p = 0.0006 versus water‐NC

Figure 5 presents the urinary excretion of the oxidative stress markers: TBARS (Figure 5a), 8‐iso‐prostaglandin F_2α,_ 8‐isoPGF_2α_ (Figure 5b), and 8‐hydroxy‐2'‐deoxyguanosine, 8‐OHdG (Figure 5c).

There was no significant difference (*p* = 0.730) between the TBARS excretion in the rats fed the NC‐diet (169 ± 5 µmol/24 h) versus those fed the HC‐diet (159 ± 5 µmol/24 h). Furthermore, neither tempol nor BSO affected the TBARS excretion in the rats fed the NC‐diet (tempol‐NC: 155 ± 7 µmol/24 h, *p* = 0.588 and BSO‐NC: 152 ± 7 µmol/24 h, *p* = 0.461) or HC‐diet (tempol‐HC: 188 ± 14 µmol/24 h, *p* = 0.4219 and BSO‐HC: 174 ± 7 µmol/24 h, *p* = 0.502).

The urinary excretion of 8‐isoPGF_2α_ in the rats fed the HC‐diet was statistically higher than NC‐diet (7.10 ± 0.19 ng/24 h vs. 5.10 ± 0.29 ng/24 h, *p* = 0.0006). Tempol did not differentially affect the rats fed the NC‐diet versus the HC‐diet in terms of the excretion of 8‐isoPGF_2α_ in the urine (tempol‐NC: 5.20 ± 0.43 ng/24 h, *p* > 0.999 and tempol‐HC: 5.56 ± 0.85 ng/24 h, *p* = 0.529). In contrast, BSO increased the excretion of 8‐isoPGF_2α_ about two‐fold in the rats fed the NC‐diet (12.31 ± 1.65 ng/24 h, *p* = 0.0006) and about 1.6‐fold in the rats fed the HC‐diet (11.16 ± 0.91 ng/24 h, *p* = 0.0131).

There was no significant difference (*p* = 0.996) in 8‐OHdG excretion between the rats fed the NC‐diet (1.92 ± 0.17 ng/24 h) and those fed the HC‐diet (1.81 ± 0.12 ng/24 h). Furthermore, neither tempol nor BSO affected the 8‐OHdG excretion in the rats fed the NC‐diet (tempol‐NC: 2.07 ± 0.13 µg/24 h, *p* = 0.986 and BSO‐NC: 2.14 ± 0.24 µg/24 h, *p* = 0.937) or HC‐diet (tempol‐HC: 1.91 ± 0.15 µg/24 h, *p* = 0.998 and BSO‐HC: 1.71 ± 0.10 µg/24 h, *p* = 0.996).

**FIGURE 6:**
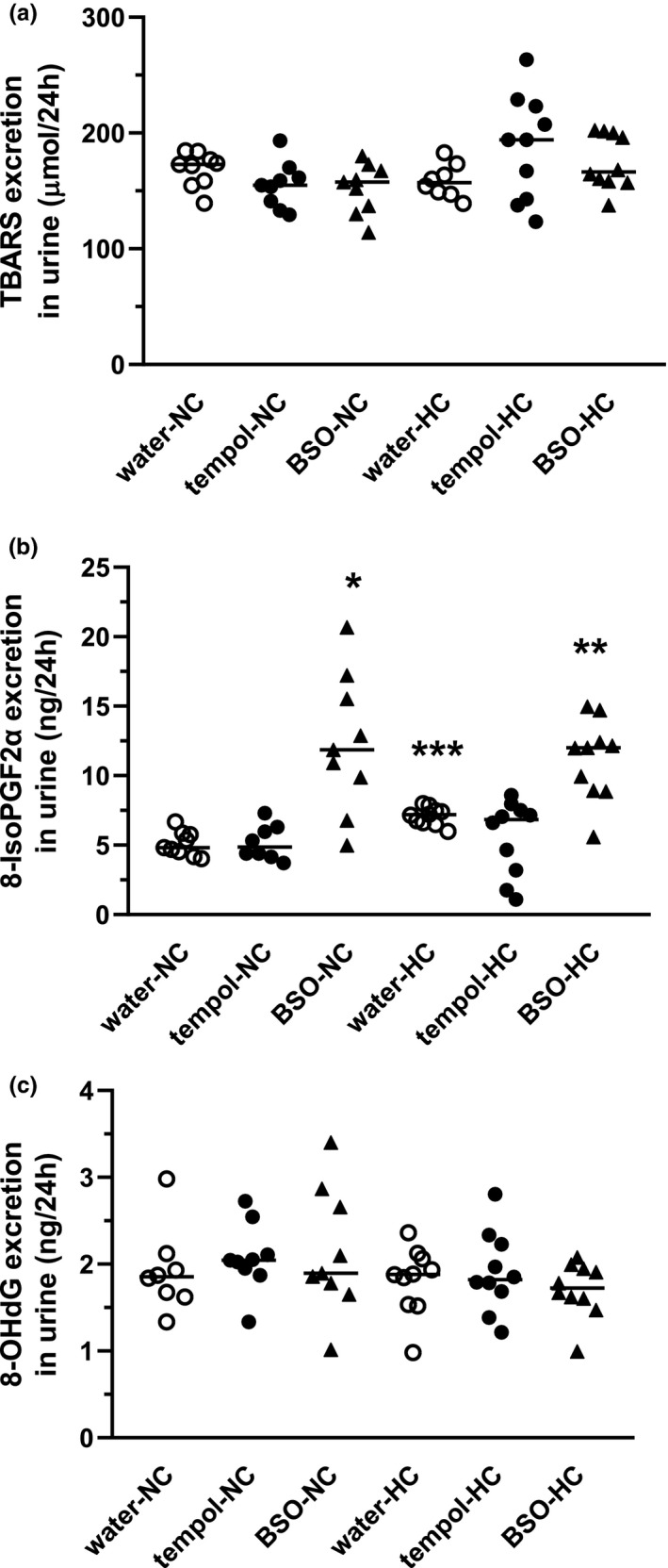
Urinary protein excretion is modified by tempol and BSO in rats fed normocholesterolemic or hypercholesterolemic diets. Rats were fed normocholesterolemic (NC) or hypercholesteremic (HC) diets over 28 days along with the oral supplementation (10 days) of water (○): 2 mM tempol (●) and 20 mM BSO (▲). Urinary excretion of protein (a), albumin (b), and nephrin (c). The results are presented as individual data points with a mean (protein) or a median (albumin, nephrin). Statistical significance was measured using a Kruskal Wallis test with a Dunn post‐hoc test (albumin: *p = 0.0143 versus water‐NC, nephrin **p = 0.0038 versus water‐HC)

6 presents the urinary excretion of total proteins (Figure 6a), albumin (Figure 6b). and nephrin (Figure 6c). The excretion of urinary protein in the rats that did not receive the administration of tempol and BSO were on comparable level (*p* = 0.228) to both the NC‐diet (water‐NC: 109 ± 5 mg/24 h) and HC‐diet groups (water‐HC: 124 ± 3 mg/24 h). The administration of tempol did not affect urinary protein excretion in the rats fed the NC‐diet (tempol‐NC: 110 ± 3 mg/24 h, *p* > 0.999) or HC‐diet (tempol‐HC: 120 ± 3 mg/24 h, *p* = 0.991). Also, BSO administration did not affect the urinary protein excretion in the rats fed the NC‐diet (BSO‐NC: 102 ± 3 mg/24 h, *p* = 0. 888) or those fed the HC‐diet (BSO‐HC: 135 ± 6 mg/24 h, *p* = 0.542).

The urinary albumin excretion (Figure 6b) in the rats on the HC‐diet was 3.6‐fold higher than that in the rats on the NC‐diet (water‐HC: 640 µg/24 h; 326–1004 µg/24 h vs. water‐NC: 188 µg/24 h; 173–325 µg/24 h, *p* = 0.0143). Neither tempol nor BSO administration affected (*p* > 0.999) urinary albumin excretion in the rats fed the NC‐diet (tempol‐NC: 187 µg/24 h; 173–290 µg/24 h and BSO‐NC: 210 µg/24 h; 188–233 µg/24 h) and HC‐diet (tempol‐HC: 685 µg/24 h; 490–1168 µg/24 h and BSO‐HC: 387 µg/24 h; 218–528 µg/24 h).

The urinary nephrin excretion values (Figure 6c) were comparable between the rats with the NC‐ and HC‐diets without the administration of tempol or BSO (water‐NC: 2.68 ng/24 h; 1.62–3.90 ng/24 h vs. water‐HC: 2.96 ng/24 h; 1.18–3.73 ng/24 h, *p* = 0.998). BSO increased the nephrin excretion in the rats fed the HC‐diet (8.19 ng/24 h; 6.85–11.40 ng/24 h, *p* = 0.0038) but not in the rats from the NC‐diet group (1.81 ng/24 h; 1.02–5.96 ng/24 h, *p* = 0.999).

## 4 DISCUSSION

The main findings of the present study are (1) diet‐induced hypercholesterolemia does not modify the action of Ap4A on CBP; (2) a superoxide dismutase mimetic, tempol, and an inhibitor of GSH synthesis, BSO, do not modify the Ap_4_A‐induced reduction of CBP in normocholesterolemic and hypercholesterolemic rats; (3) tempol abolishes the late phase of the Ap_4_A‐induced increase in CBP in normocholesterolemic rats and the early and late phases of the Ap_4_A‐induced increase in CBP in hypercholesterolemic rats; and (4) BSO abolishes the late phase of the Ap_4_A‐induced increase in CBP in normocholesterolemic rats.

Using laser Doppler flowmetry, we have investigated changes in CBP, MBP in response to the intravenous infusion of Ap_4_A, which induced bidirectional changes in CBP, namely a sharp decrease recorded over a period of up to 2 min and also a sharp increase over the next 2 min followed by a gradual decline; however, the CBP still remained statistically significantly higher than the basal values between 20 and 30 min after the start of the Ap_4_A infusion in the normocholesterolemic rats. Moreover, Ap_4_A induced a rapid decrease in MBP recorded over a period of up to 2 min in the normocholesterolemic rats but the rising phase in MBP was not observed. However, it must be mentioned that studies on renal MBP and data analysis are complicated by the complex vascular anatomy of the region (Pallone et al., 2003). Moreover, we are aware that relative small number of rats used to study the Ap_4_A‐induced changes of renal v limits the interpretation of our result. However, it seems that even taking into account these limitations the obtained results can be considered with data provided by other researchers. It has been shown that tempol increases renal MBP but superoxide dismutase inhibitor, diethyldithiocarbamate, reduces renal MBP suggesting that superoxide produced in renal medulla may play role in regulation of blood perfusion in this renal region (Zou et al., 2001). With these results in mind, we conducted further experiments, the results of which indicated the importance of redox balance in diet‐induced hypercholesterolemic rats regarding the Ap_4_A‐induced regulation of CBP and MBP and glomerular injury.

The kidney vasculature is an important target for hypercholesterolemic‐induced alternations in vasomotor activity and structure. Our previous study provided evidence that Ap_4_A‐induced cortical vasoconstriction mediated by P2Rs and adenosine A1 receptors may also be involved in the Ap_4_A‐induced increasing phase of renal blood perfusion in normocholesterolemic rats (Kreft et al., 2020). This observation is consistent with our earlier finding regarding the Ap_4_A‐induced contraction of the glomerular microvasculature (Szczepanska‐Konkel et al., 2005). In our present study, we have shown that the vasoconstrictor and vasorelaxation reactivity of the cortical and medullary vasculatures were not affected by hypercholesterolemia induced by 28 days of feeding with a HC. Moreover, the Ap_4_A‐induced changes in CBP about 31%–37% of the basal values in both the NC‐diet and HC‐diet groups. However, it has been shown that a long‐lasting HC in pigs resulted in inflammation‐based cortical neovascularization, the increase in vascular permeability, and the abolition of renal blood flow to acetylcholine (Chade et al., 2007). There is also evidence that altered vasomotor regulation by the endothelium in hypercholesterolemia is an effect of decreased nitric oxide availability in the renal circulation and renal artery (Feldstein et al., 1999; Stulak et al., 2001). Moreover, published data suggests that the abnormal vascular response to acetylcholine may represent a defect in endothelial vasodilator function because the paradoxical vasoconstriction induced by acetylcholine was observed in patients with coronary atherosclerosis (Ludmer et al., 1986). Importantly, the chronic antioxidant supplementation may normalize the endothelium‐dependent relaxation by decreased oxidation of LDL and by increasing bioavailability of nitric oxide in hypercholesteremic pigs (Stulak et al., 2001). It is worth to mention that the NO‐induced endothelium‐dependent vasorelaxation is dependent on activation of soluble guanylyl cyclase in the vascular smooth muscle cells (Wennysia et al., 2021). It has been shown that pharmacological activation of this enzyme restores renal blood flow and increases arterial blood pressure in NO‐deficient animals (Dautzenberg et al., 2014). Thus, the disturbances in regulation of CBP and MBP in hypercholesterolemic rats may be result of endothelial and/or vascular smooth muscle cells dysfunction. It seems possible that, in our model of 28 days of diet‐induced hypercholesterolemia in Wistar rats, the influence of endogenous oxidative stress could have been barely noticed.

Among the investigated markers of oxidative stress, we found significant differences for 8‐isoPGF_2α_ between NC and HCs, which indicates the development of oxidative stress in hypercholesterolemic rats. However, the serum concentration of oxLDL was not changed in rats on a HC which may indicate some limitations of the experimental model. However, at the same time, we have found that animals on a 28‐day HC are characterized by increased urinary albumin excretion, which indicates that even short‐term hypercholesterolemia negatively affects the glomerular filter permeability to albumin and may predispose to organ damage in the presence of overlapping other adverse conditions. Thus, in addition to using a diet with a higher cholesterol content, the in vivo redox balance in the body was modified by the administration of a DL‐buthionine‐S,R‐sulfoximine (BSO)‐specific and irreversible inhibitor of γ‐glutamyl‐cysteine synthetases (a rate‐limiting enzyme catalyzing the synthesis of GSH and acting as a free radical scavenger) and tempol (membrane‐permeable, metal‐independent superoxide dismutase mimetic) (Griffith & Meister, 1979; Xiao & Loscalzo, 2020). In the present study, actual BSO dose levels calculated from the amount of water drunk on average (314 mg/kg/day [NC‐diet] and 378 mg/kg/day [HC‐diet]) decreased the plasma GSH concentrations by about 50% in the NC‐ or HC‐diets. On the other hand, tempol (26 mg/kg/day in NC‐diet group, 24 mg/kg/day in HC‐diet group) did not affect the plasma GSH concentrations in the rats fed the NC‐ and HC‐diets.

Damages caused by ROS/RNS include structural and functional modifications of the cellular constituents, such as membranous lipids, proteins, and DNA; thus, the methods for measuring the levels of in vivo oxidative stress are based on detecting the changes in the oxidation products of endogenous molecules level. Lipid peroxidation is one of the indices of oxidative stress and has been implicated as a contributing factor in a range of vascular dysfunctions. The markers used to measure oxidative stress in vivo are the arachidonic acid oxidation products, but 8‐iso‐prostaglandin F_2α_ (8‐iso‐PGF_2α_) is most often measured. The excessive generation of 8‐iso‐PGF_2α_, exerting biological effects exclusively via the activation of the TxA_2_ prostanoid receptor (Bauer et al., 2014), has been attributed to chemical lipid peroxidation and prostaglandin‐endoperoxide synthases‐mediated enzymatic lipid peroxidation (Erve et al., 2015). In our experiments, HC‐diet alone, lasting 28 days, increased about 40% urinary excretion of 8‐isoPGF_2α_ suggesting the development of oxidative stress. Furthermore, BSO increased the urinary excretion of 8‐isoPGF_2α_ in rats fed the NC‐diet (two‐fold) and HC‐diet (1.6‐fold). Interestingly, evidence suggests that 8‐isoPGF_2α_ affects glomerular function (Takahashi et al., 1992), thus, urinary excretion of 8‐isoPGF_2α_ is not only a marker for lipid peroxidation in vivo but 8‐isoPGF_2α_ may also participate as mediator of renal oxidant injury. Our present results are in line with the results of other studies in which the urinary excretion of 8‐isoPGF_2α_ was found to be abnormally elevated in the vast majority of hypercholesterolemic patients due to increased F2‐isoprostane formation, which is, in part, related to high LDL levels. Furthermore, vitamin E supplementation was associated with reduction in urinary 8‐isoPGF_2α_ excretion (Davi et al., 1997).

Hypercholesterolemia increases ROS level in kidney and is associated with the formation of the oxidized low‐density lipoprotein, oxLDL (Kumar et al., 2006). In our experiments, using an ELISA‐based measurement (ELISA kit for rat oxLDL, Biorbyt, orb411300), we did not see a statistical significant difference between the oxLDL concentration in the serum of rats fed the NC‐ and HC‐diets. Moreover, we did not note any effects of BSO on the oxLDL concentration. It should be noted that there may have been be methodological problems related to the measurement of serum oxLDL concentration due to insufficient antibody specificity (Friedman et al., 2002) and low concentration of oxLDL, which could have constituted 0.001%–5% of LDL (Itabe & Ueda, 2007). Using antibodies recognizing oxidized phosphatidylcholine, including adducts with proteins that do not exhibit an apoB enhanced level of oxLDL, was reported in patients with coronary artery disease (Ehara et al., 2001). Moreover, the rapid taking up of oxLDL by Kupffer cells, which may contribute to keeping the concentration of oxLDL as low as possible under a given (pato)physiological condition, should be noted (Van Berkel et al., 1991). Furthermore, the mechanisms of oxLDL taking up may be activated in pathophysiological conditions; the experiments performed on mice given BSO at an average dose of 56 mg/kg/day provides evidence that the inhibition of GSH synthesis induces macrophage receptors for oxLDL and enhances its uptake (Yang et al., 2015). Also, it has been demonstrated that autoantibodies against oxLDL are present in both rabbit and human sera (Palinski et al., 1989).

Another marker of lipid oxidation is malondialdehyde (MDA) concentrations in the serum. It is one of the best studied end‐products of the peroxidation of polyunsaturated fatty acids in clinical samples using TBARS; however, it should be noted that a TBARS assay measures the MDA generated by the decomposition of lipid peroxides in biological samples rather than the free MDA, and aldehydes other than MDA may also react with TBARS (Ito et al., 2019). In our experiments, the urinary excretion of TBARS was not affected by diet nor different cholesterol and BSO concentrations.

ROS attacking DNA produces a large number of purine and pyrimidine‐derived lesions. The most studied biomarker is 8‐hydroxy‐2'‐deoxyguanosine (8‐OHdG), a stable end product of nonenzymatic DNA oxidation excreted into the urine. Under physiological conditions, the level of urinary 8‐OHdG correlates with the metabolic rate (Shigenaga et al., 1989), may be increased in situations of endogenous or pharmacological‐based depletion of cellular GSH involved in the process of DNA repair (Bansal & Simon, 2018; Graille et al., 2020). Moreover, urinary 8‐OHdG levels were validated as a sensitive biomarker of oxidative stress in an animal model using the administration of xenobiotics (Kadiiska et al., 2005). In our study, we did not observe significant effects of a HC‐diet and BSO on the urinary excretion of 8‐OHdG, suggesting that BSO at the dose used is not genotoxic.

Tempol and BSO in our in vivo measurements did not affect Ap_4_A‐induced decreased CBP in either the normocholesterolemic or hypercholesterolemic rats, suggesting that the superoxide anion is probably not involved in the Ap_4_A‐induced contraction of the cortical vasculature. In contrast, it has been shown that in vitro‐produced superoxide stress resulted in a decrease in the Ap_4_A‐stimulated contractility of the guinea pig vas deferens (Al‐Rawi et al., 2008). Further, another dinucleoside polyphosphate, uridine adenosine tetraphosphate (Up_4_A), was found to involve P1 and P2X receptors in concentration‐dependent contractions, and this effect was found to be significantly reduced via treatment with tempol (Linder et al., 2008). On the other hand, the observation that tempol affects the Ap_4_A‐induced increase in CBP suggests the involvement of a superoxide anion in this process. Analysis of data of NC‐ and HC‐diets points to the possibility that the role of the superoxide anion is more pronounced in hypercholesterolemic rats, in which the tempol scavenging of the superoxide anion prevents both the early and late phases of cortical vasculature responses, than in normocholesterolemic rats, in which tempol only prevents the early phase of the cortical vasculature response. In the endothelium, the superoxide anion interacts with nitric oxide, modulating its bioavailability and vasorelaxation potential. It seems important that hypercholesterolemia is accompanied by decreased nitric oxide and increased superoxide anion production (Feron et al., 1999; Ohara et al., 1993). The superoxide anion reacting with nitric oxide produces a peroxynitrite anion, which, in turn, will promote oxidation and nitration reactions affecting different biomolecules (Radi, 2018). Under physiological conditions, there is competition between peroxynitrate formation and superoxide dismutation; thus tempol restores the NO bioavailability due to the scavenging of superoxide anion to oxygen and peroxide hydrogen, which is further efficiently scavenged by catalase. It has been shown that tempol enhances the catalase activity and prevents oxidative stress induced by peroxide hydrogen (Krishna et al., 1996). However, peroxide hydrogen may activate cGMP‐dependent protein kinase or form a hydroxyl radical, the most powerful oxidizing intermediate (Radi, 2018). Moreover, we have previously shown that cGMP‐dependent protein kinase is involved in the relaxation of isolated renal glomeruli induced by agonists of P2‐recpetors (Kasztan & Jankowski, 2016). Bearing in mind the above mechanisms of tempol action, the NO‐independent effect of tempol should also be taken into account. It has been shown that acute administration of tempol caused a fall in sympathetic nervous system activity that was not prevented by NO synthase inhibition (Xu et al., 2001). These findings indicate regulation of renal perfusion is complex and also related on renal sympathetic nerve activity.

We observed that oxidative stress produced by BSO results in the prevention of the Ap_4_A‐induced late phase increase in CBP in normocholesterolemic rats but not in hypercholesterolemic rats. These observations, at least in part, are in agreement with published data showing that BSO causes a decreased response to the endothelium‐independent vasorelaxants in thoracic aortic rings isolated from rats fed a standard diet (Banday et al., 2007). Taken together, in terms of the observation that the late phase is prevented by tempol but not BSO, it seems that this observation might be due to the superoxide anion‐mediated action of Ap_4_A. Unfortunately, at the moment, we cannot identify an explanation for the observations regarding the retained late‐phase response in rats fed a HC‐diet supplemented with BSO.

The total proteins excretion in the urine was not affected by the HC‐diet or oxidative stress modulators in our experiments. However, urinary albumin excretion in the rats fed the HC‐diet was 3.6‐fold higher than in those fed the NC‐diet, but BSO did not enhance the level of albumin excretion in either group of rats. On the other hand, the administration of tempol did not significantly reduce the severity of albuminuria in the hypercholesterolemic. The increased albumin excretion observed in the HC‐diet‐fed rats, along with simultaneous elusive changes in the excretion of oxidative stress markers, suggests that albuminuria may be taken as an early marker of glomerular dysfunction accompanying hypercholesterolemia. Increased albumin excretion in urine may be the result of enhanced glomerular permeability to albumin and/or the reduced renal uptake of albumin by proximal tubules in hypercholesterolemic rats (Sandoval et al., 2012). It has been shown that rats with diet‐induced hypercholesterolemia develop renal interstitial fibrosis (Eddy, 1996), which may affect tubular function. Therefore, the potential involvement of proximal tubule processes in albuminuria observed in rats fed HC‐diet merits further studies including the analysis of urinary excretion of proximal reabsorption markers (e.g. megalin or β2‐microglobulin). On the other hand, glomerular permeability to albumin depends on the hemodynamic conditions in the glomerular capillaries and on the three‐layer structure of the filtration barrier consisting of endothelial cells (inner layer), the basement membrane, and podocytes (outer layer). The role of hemodynamic conditions has been shown in micropuncture experiments performed in rats fed a HC‐diet demonstrated the elevation of colloid osmotic pressure associated with an increase in intraglomerular pressure (Kasiske et al., 1990). It was also found that endothelial permeability to albumin was greater in hypercholesterolemic pigs than in those on a normal diet (Lamack et al., 2006). There is evidence that both endotheliocytes and podocytes are capable of producing and utilizing ROS/RNS, and balance between these processes affects glomerular permeability to albumin (Gwinner et al., 1998). Nitric oxide as well as tempol preserves the glomerular permeability to albumin by antagonizing superoxide anion (Duann et al., 2006; Sharma et al., 2005). Also, it has been observed that the renal nitric oxide synthesis is reduced in the AT1‐receptor sensitive pathway, leading to podocyte stress and proteinuria in rats fed a HC‐diet (Attia et al., 2002, 2004). Injury of podocytes may be assumed based on increased amounts of podocyte specific proteins in the urine. In our experiments, we used nephrin, a key protein of the outer layer of the glomerular barrier (Li et al., 2015). We did not detect significant changes in nephrin excretion between the urine of the rats fed a NC‐ versus HC‐diet; however, additional BSO‐induced oxidative stress led to significant nephrinuria, reflecting an irreversible podocyte injury and reduction their number in glomeruli.

## 5 CONCLUSIONS

Our results suggest the important role of redox balance in the extracellular nucleotide regulation of the renal vasculature as well as glomerular injury. It has been suggested that free radicals are involved in the vasorelaxant response to P2‐receptor agonist, both in normocholesterolemia and hypercholesterolemia. It seems that disturbances in the production of free radicals in hypercholesterolemic organism may lead to changes in the microcirculation of the kidneys contributing to the development of chronic disorders of this organ function. In addition, hypercholesterolemia that increases urinary albumin excretion may contribute to the progression of chronic kidney disease.

## CONFLICT OF INTEREST

The authors declare no conflicts of interest.

## AUTHOR CONTRIBUTION

DK, JM conceptualized and designed the experiments. DK, KE, SZK, CG performed experiments. DK, KA collected and analyzed the data. KA, JM contributed to data interpretation and statistical analysis. JM wrote and edited the manuscript. All authors approved the final manuscript.
